# New Books

**Published:** 2009-11

**Air Quality in Urban Environments**

Ronald E. Hester, Roy M. Harrison, eds.

New York:Springer, 2009. 148 pp. ISBN: 978-1-84755-907-4, $119

**Catastrophe in the Making: The Engineering of Katrina and the Disasters of Tomorrow**

William Freudenberg

Washington, DC:Island Press, 2009. 224 pp. ISNB: 978-1-59726-682-6, $26.95

**Chasing Molecules: Poisonous Products, Human Health, and the Promise of Green Chemistry**

Elizabeth Grossman

Washington, DC:Island Press, 2009. 288 pp. ISBN: 978-1-59726-370-2, $26.95

**Climate Change in Eurasian Arctic Shelf Seas: Centennial Ice Cover Observations**

I.E. Frolov,, Z.M. Gudkovich, V.P. Karklin, E.G. Kovalev, V.M. Smolyanitsky

New York:Springer, 2009. 166 pp. ISBN: 978-3-540-85874-4, $129

**Corporate Power in Global Agrifood Governance**

Jennifer Clapp, Doris Fuchs, eds.

Cambridge, MA:MIT Press, 2009. 312 pp. ISBN: 978-0-262-01275-1, $48

**Ensuring the Integrity, Accessibility, and Stewardship of Research Data in the Digital Age**

Committee on Ensuring the Utility and Integrity of Research Data in a Digital Age, National Academy of Sciences

Washington, DC: National Academies Press, 2009. 152 pp. ISBN: 978-0-309-13680-8, $36.95

**Ethics Education and Scientific and Engineering Research: What’s Been Learned? What Should Be Done?**

Rachelle Hollander, Carol R. Arenberg, eds.

Washington, DC:National Academies Press, 2009. 58 pp. ISBN: 978-0-309-14001-0, $21

**Extinction in Our Times: Global Amphibian Decline**

James P. Collins, Martha L. Crump

New York:Oxford University Press, 2009. 304 pp. ISBN: 978-0-19-531694-0, $29.95

**Hormones and Pharmaceuticals Generated by Concentrated Animal Feeding Operations: Transport in Water and Soil**

Laurence S. Shore, Amy Pruden, eds.

New York:Springer, 2009. 138 pp. ISBN: 978-0-387-92833-3, $129

**Impacts of Point Polluters on Terrestrial Biota**

Mikhail Kozlov, Elena Zvereva, Vitali Zverev

New York:Springer, 2009. 466 pp. ISBN: 978-90-481-2466-4, $169

**Learning Science in Informal Environments: People, Places, and Pursuits**

Philip Bell, Bruce Lewenstein, Andrew W. Shouse, Michael A. Feder, eds.

Washington, DC:National Academies Press, 2009. 352 pp. ISBN: 978-0-309-11955-9, $49.95

**Nanotechnology: Consequences for Human Health and the Environment**

Ronald E. Hester, Roy M. Harrison, eds.

New York:Springer, 2009. 134 pp. ISBN: 978-1-84755-956-2, $79.95

**Outbreak Investigations Around the World: Case Studies in Infectious Disease Field Epidemiology**

Mark Dworkin

Boston:Jones & Bartlett, 2010. 456 pp. ISBN: 978-0-7637-5143-2, $64.95

**Radioactive Particles in the Environment**

Deborah H. Oughton, Valery Kashparov

New York:Springer, 2009. 279 pp. ISBN: 978-90-481-2947-8, $189

**Studies on Science and the Innovation Process**

Nathan Rosenberg, ed.

Hackensack, NJ:World Scientific, 2009. 428 pp. ISBN: 978-981-4273-58-9, $65

**Sustainability 2009: The Next Horizon**

Imre Hronzsky, Gordon L. Nelson, eds.

New York:Springer, 2009. 245 pp. ISBN: 978-0-7354-0694-0, $139

**Sustaining the World’s Wetlands: Setting Policy and Resolving Conflicts**

Richard Smardon

New York:Springer, 2009. 325 pp. ISBN: 978-0-387-49428-9, $74.95

**The Comet Assay in Toxicology**

Alok Dhawan, Diana Anderson, eds.

New York:Springer, 2009. 461 pp. ISBN: 978-0-85404-199-2, $249

**The Disposal of Activated Carbon from Chemical Agent Disposal Facilities**

Committee to Examine the Disposal of Activated Carbon from the Heating, Ventilation, and Air Conditioning Systems at Chemical Agent Disposal Facilities, National Research Council

Washington, DC:National Academies Press, 2009. 86 pp. ISBN: 978-0-309-13818-5, $21

**The Nile: Origin, Environments, Limnology and Human Use**

Henri J. Dumont, ed.

New York:Springer, 2009. 818 pp. ISBN: 978-1-4020-9725-6, $209

**The Paradox of Scientific Authority: The Role of Scientific Advice in Democracies**

Wiebe E. Bijker, Roland Bal, Ruud Hendriks

Cambridge, MA:MIT Press, 2009. 232 pp. ISBN: 978-0-262-02658-1, $32

**The Rising Sea**

Orrin Pilkey, Rob Young

Washington, DC:Island Press, 2009. 224 pp. ISBN: 978-1-59726-191-3, $25.95

**The Yellow River: Water and Life**

Tetsuya Kusuda, ed.

Hackensack, NJ:World Scientific, 2009. 300 pp. ISBN: 978-981-4280-95-2, $82

**Toward Sustainable Communities, 2nd ed.: Transition and Transformations in Environmental Policy**

Daniel A. Mazmanian, Michael E. Kraft, eds.

Cambridge, MA:MIT Press, 2009. 352 pp. ISBN: 978-0-262-51229-9, $25

**Understanding Environmental Health: How We Live in the World**

Nancy Irwin Maxwell

Boston:Jones & Bartlett, 2009. 378 pp. ISBN: 978-0-7637-3318-6, $72.95

**Urban Health: Readings in the Social, Built, and Physical Environments of U.S. Cities**

H. Patricia Hynes, Russell Lopez

Boston:Jones & Bartlett, 2009. 302 pp. ISBN: 978-0-7637-5245-3, $61.95

**WHO Handbook on Indoor Radon: A Public Health Perspective**

WHO International Radon Project

Annapolis Junction, MD:WHO Press, 2009. 108 pp. ISBN: 978-92-4154-767-3, $30

**Women’s Global Health and Human Rights**

Padmini Murthy, Clyde Landord Smith

Boston:Jones & Bartlett, 2010. 385 pp. ISBN: 978-0-7637-5631-4, $72.95

